# Steering of Crystal Cell Volumes in Apatites and Bioapatites

**DOI:** 10.3390/molecules31040707

**Published:** 2026-02-18

**Authors:** Andrzej Kuczumow, Agnieszka Lasota, Mieczysław Gorzelak, Paulina Wojtyła-Buciora, Przemysław Biliński, Małgorzata Bernatek, Karolina Turżańska, Jan Olszewski, Przemysław Dyndor, Maciej Jarzębski, Marek Wieruszewski, Mirosław Jabłoński

**Affiliations:** 1 Faculty of Medicine and Health Promotion, The President Stanisław Wojciechowski University of Kalisz, 62-800 Kalisz, Poland; andrzej.kuczumow@gmail.com (A.K.); p.wojtyla-buciora@uniwersytetkaliski.edu.pl (P.W.-B.);; 2 Department of Jaw Orthopaedics, Medical University of Lublin, 20-093 Lublin, Poland; agnieszka.lasota@umlub.edu.pl; 3 Department of Orthopaedics and Rehabilitation, Medical University of Lublin, 20-059 Lublin, Poland; b.leszczynska@umlub.pl (M.G.); karolina.turzanska@umlub.pl (K.T.); miroslaw.jablonski@umlub.pl (M.J.); 4 Department of General and Neural Rehabilitation, Lublin Military Hospital, 20-049 Lublin, Poland; 5 Department of Physics and Biophysics, Poznań University of Life Sciences, 60-637 Poznań, Poland; 6 Department of Wood-Based Materials, Faculty of Forestry and Wood Technology, Poznań University of Life Sciences, Wojska Polskiego 28, 60-627 Poznań, Poland; marek.wieruszewski@up.poznan.pl

**Keywords:** apatite crystal volumes, energetics of apatite/bioapatite crystals, ΔE vs. Δd diagrams, Braggs’ dimensions, reformulation of Braggs’ law

## Abstract

The biological variability of apatites in different hard tissues of organisms was the starting point for this investigation. Materials such as whale rostrums, ganoine, and some fish bones were analyzed. It has been proven that different organisms select specific kinds of apatites for the construction of their hard organs at the level of the crystal cell. This probably results from the long-lasting adaptation of the construction to their environmental needs. The materials are characterized by the parameters Δd and ΔE, being the real and apparent deviations from Bragg’s dimension d and the energy of excitation in XRD—E. This study is based on previously published, verified results from a number of researchers and research groups. The derivation of expressions was possible due to an original approach to Bragg’s equation, finally finished in the reformulation of the law, which describes the interplay between the absolute value of the probing excitation energy E and the crystal cell’s internal volume V. It enabled the classification of apatite biomaterials in living and fossil organisms, as well as the classification of the apatite excretions. In addition, the following different possible modes of changes in Bragg’s dimension d were illustrated—spontaneous geometrical expansion, thermal action, pressurization, and single- and multiple-ion exchanges. The contributions of such expansions were estimated. We can steer the cell volume of apatites in various ways. It has been proven that the volume expansion is linearly coupled with the expansion of Bragg’s d parameter in the hexagonal system.

## 1. Introduction

There are many different hard tissues supported by apatites and functioning in nature [1]. They belong to the category of composite compounds, since they always compose smaller (enamel, rostra [2,3]) or greater (dentin, bone [4]) parts of different organic materials [5]. Due to their significance in the normal existence and functioning of organisms, they were extensively studied to explain their features [6]. One must realize that organs containing bioapatite have distinct anatomical meanings and functions (teeth, bones, tusks, femurs, antlers, rostra, bulla), and sometimes it is difficult to find a common benchmark when considering them, perhaps aside from the apatite presence. Some reviews of more exotic members of the family of hard tissues were written by Schultze [7] and Kawasaki et al. [8]. A useful review of extra-skeletal bones in mammals was provided by Nasoori [1]. A not-unimportant reason for our contribution is that the obtained knowledge could be applied in potential syntheses of new biomaterials, including ones with biomimetic reactions. Another reason for our interest is the fact that some animal wastes can be used to produce nanosized bioapatite particles on a laboratory or industrial scale [9]. There are some very interesting reviews of various biomaterials, which help provide a better understanding of them. Unfortunately, the studies mostly focus on either the morphological characteristics of tissues or crystallites [10] or on comparisons of the mechanical features of the considered substances [11,12], rather than on chemical and crystallographic parameters. The latter features seem to determine the whole rest of the behaviour of a given organ, which is not a common opinion. The apatite behaviour at the crystallographic-cell level is a basis of all the further characteristics of a given biomaterial in this text.

Since it concerns the subject of our contribution, it is important to mention the tissues under scrutiny: bone, enamel, and dentin of mammals; deer antlers; ivory of elephants and mammoths; similar tusks of walruses; rostra and bullae of, e.g., whales; enameloids of fish; carapaces of tortoises; and other similar tissues. These are characterized by the presence of apatite crystallites as the main hard components, admixed with organic matter, often but not exclusively collagenous. Given the contents, the organic part can be treated as a matrix (dentin, bone) or as a minor but still important internal component (enamel, rostra). In fact, none of the above materials are pure mineral hydroxy-, chlor- or fluorapatite, but they are all composite materials. Even if we limit consideration to the mineral parts of the tissues, we must be aware that the said apatites are enriched in sometimes considerable amounts of additional ions, such as magnesium, sodium, carbonate, and fluorine [13], and depleted of hydroxyl entities [14]. This indicates that, each time, the apatite part is different both from the original hydroxyapatite and from the apatites in the other organs of one organism (e.g., bone apatite differs from enamel and dentin apatites) and is different temporarily—ontogenetically (e.g., young and mature bones differ from each other)—and is different among species—phylogenetically (e.g., dentin apatite of mammals is still a different material than ivory apatite from elephants or mammoths). Nevertheless, similarities were also observed—Li et al. [15] suggested that whale rostrum is, on the one hand, similar, due to its great density, to enamel, while on the other hand, due to its morphological features, it is similar to very dense bone, much depleted of collagen. It is astonishing that, although scientists have studied the shapes and dimensions of apatite crystallites [4], the kinds of organic matrices, the morphology of materials, and the mechanical features of them [16], they somewhat neglected comparative studies of the single-crystallographic cells of particular apatites contributing to different organisms. Perhaps the studies on human bones and teeth are here the exceptions. Such single-crystallographic cells are, in fact, very important. We have proven in our previous contributions that only the apatite and bioapatite cell parameters determine the location of apatite within the isomorphic series of compounds [17]. This seems to involve the given bioapatite inside the whole series of bioapatites and apatites. It allows one to see clearly the ordering of all the apatites, without prejudging whether the mutual transformation of one shape into another is at all possible. One can also set the standard point, from which all the series will be considered, and the hydroxyapatite plays this role in our text. The comfortable comparative knowledge can be obtained using the special set of equations describing the apparent energetic changes in apatite cells, resulting from the changes in Braggs’ dimension d:

∆E = −6.2 × ∆d/(d^2^ × sin θ) (1a)

and, optionally,

ΔE = −(1/6.2) × Δd × E^2^ × sin θ (1b)

or an equivalent one:

∆E = k∆d(1c)

If we compare Equations (1a) and (1b) with (1c), respectively, the relevant values for coefficient k are as follows:

K_1_ = −6.2/(d^2^sin θ) (2a)

and

K_2_ = −(1/6.2) * E^2^sin θ (2b)

where ∆E expressed in keV is the energy difference resulting from the change in Bragg’s crystallographic dimension ∆d in angstroms Å, occurring under the influence of external radiation, leading to the transformation (sometimes very small) of one apatite material into another one. The K coefficient determines the compound’s membership in the given isomorphic series of chemicals. Those K coefficients have a great significance, since they determine the location of the points within the hexagonal structure, as reflected in the expansion expressed by changes in sin θ, i.e., they reflect purely geometrical swelling or squeezing. Although the energy of the X-ray tube is given in keV, we often recalculate our energy results in eV through multiplication to obtain more convenient units.

One can add several equivalent equations to the above ones:

ΔE = (6.2/d_1_)(1/sin θ_1_ − sin θ_2_) (1d)

ΔE = (6.2/sin θ_1_)(1/d_2_ − 1/d_1_) (1e)

ΔE = −6.2 × Δ(sin θ)/(d × sin^2^ θ) (1f)

All of them were originally derived in our previous papers.

We had the following aims to achieve in our present contribution: (i) To what level is the Bragg’s dimension of crystals characteristic of the given biomaterial of some organisms? This question can be inverted—Do the organisms choose the crystals of the selected Bragg’s dimension for their hard tissues in a way specific to given species? (ii) What are the possibilities of nature changing the Bragg’s dimensions and probing energies of excitation for bioapatites and apatites in general? (iii) Is the dimension d joined quantitatively with the volume of the crystallographic cell? (iv) Can we reformulate Bragg’s law in terms of other variables?

## 2. Results

### 2.1. Range of ΔE–Δd Diagrams for Apatites and Bioapatites

**Figure 1:**
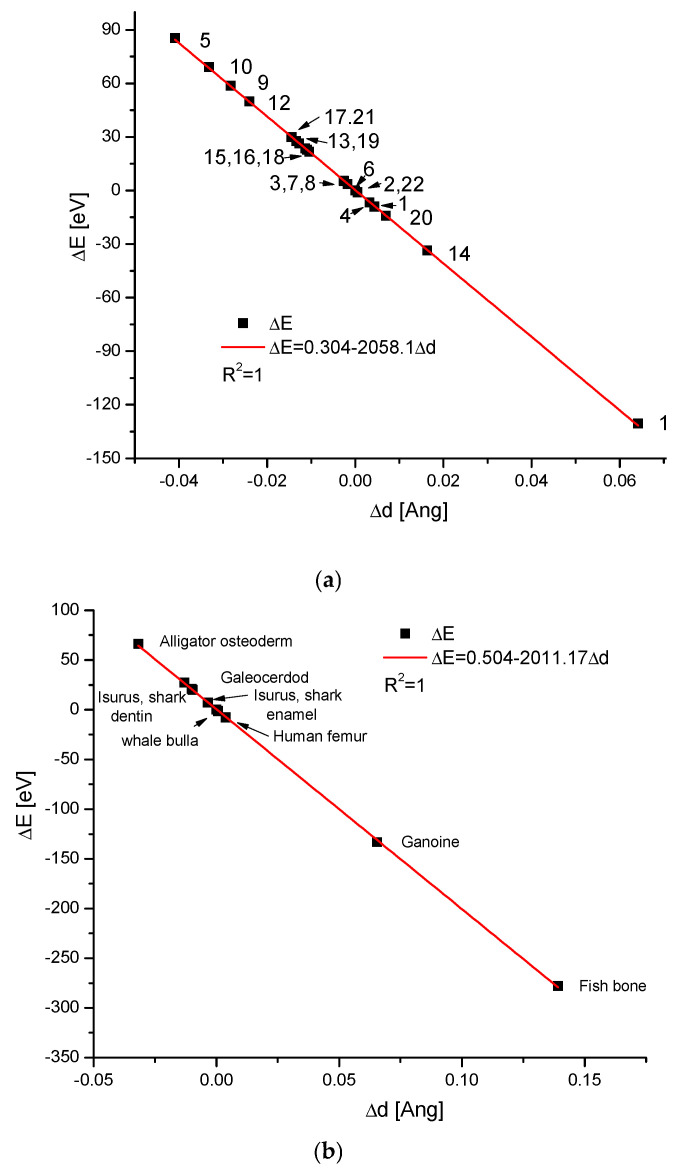

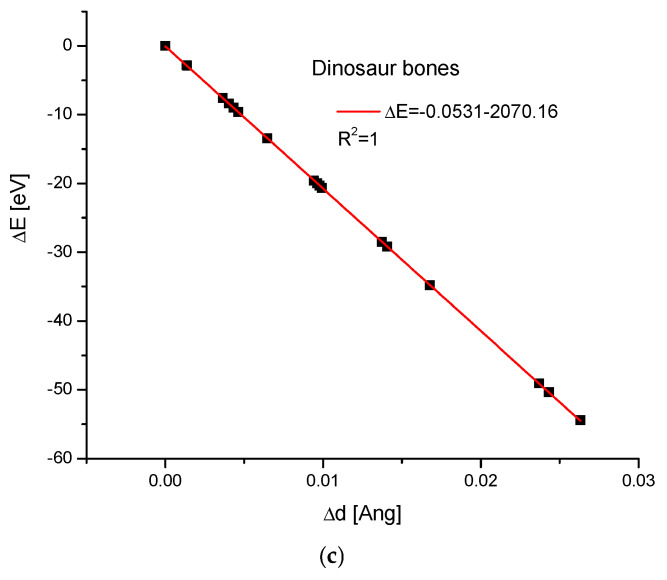
(**a**) ΔE–Δd diagram, for basic apatite forms occurring in the organs of different animals and popular geological/synthetic ones. The numbers stand for: 1—human bone; 2—hydroxyapatite—this is the standard point; 3—human enamel [19]; 4—human dentin [20]; 5—whale rostrum [3]; 6—ivory [21]; 7—mammoth ivory [22]; 8—kidney apatite-type stone [23,24,25,26]; 9—alligator osteoderm [27]; 10—ganoine [28]; 11—elk antler [29]; 12—antler cortical; 13—antler trabecular [30]; 14—*Isurus oxyrinchus*—enameloid; 15—*Isurus oxyrinchus*—dentin; 16—*Galeocerdo cuvier*—enameloid; 17—*Galeocerdo cuvier*—dentin [31]; 18—geological fluorapatite [32]; 19—synthetic hydroxyapatite; 20—synthetic fluorapatite single crystal [33]; 21—geological hydroxyapatite; (**b**) the remaining results, with the spectacular data for the ganoine and some extremal fishbones [34,35]. It is a small number among all available representative sets of samples. See, please, that the biomaterials are considered as uniform entities, without splitting into potential constituents [36], inorganic or organic. (**c**) The same is true for the dinosaur bones, as a typical fossil material composed of francolite, according to Piga et al. [37,38], showing that even diagenetic changes in bones obey the rules, meaning that they belong to one isomorphic family. Please compare the coefficient −2070.16 for the Spain dinosaur francolite-based series with the theoretical value for the purely geometrical expansion of the apatites, −2068.9.

We selected a large amount of available information on the bioapatites present in the different organs of many animals. It is presented in Figure 1a, where we show the points on the diagram ΔE–Δd. Due to the large number of the points, we designed them with the numbers explained in the caption. Some geological and synthetic apatites, fossils, and calculi [18] were introduced for comparison.

This is not the only diagram concerning apatite biomaterials. Some remaining results are shown in Figure 1b. We know many other measurement results for other bioapatite samples, but they lie along the same lines. The range of specific points covers the energy values +100/−300 eV, with Δd equivalents −0.014/+0.14Å. Nevertheless, the clear densification of points lies in the region −50/+60 eV, indicating that the majority of organisms use only particles within a relatively narrow range of the parameters. The ranges lie on both sides of the hydroxyapatite position. Perhaps the main conclusion derived from the above diagrams is that animals use apatite cells with quite strictly defined parameters for themselves, and selection occurs at the level of single apatite cells, not the crystallites, as previously suggested [39,40]. Thus, the parameters of the single-crystal cell of bioapatite can uniquely determine the organ of a given species. We suppose that the assignment of the special type of apatite to a given species occurred during long-lasting adaptation in the processes of evolution, as was suggested by Weiner and Wagner, although they considered this question from quite a different point of view [41]. Please note that the densification of apatites is accompanied by alkalization during synthesis, while erosion and decay occur with acidification in further life [42]. Such environmental conditions can influence the crystal cell parameters. However, we must remember the measurement errors and the possible contamination of the samples.

**Figure 2:**
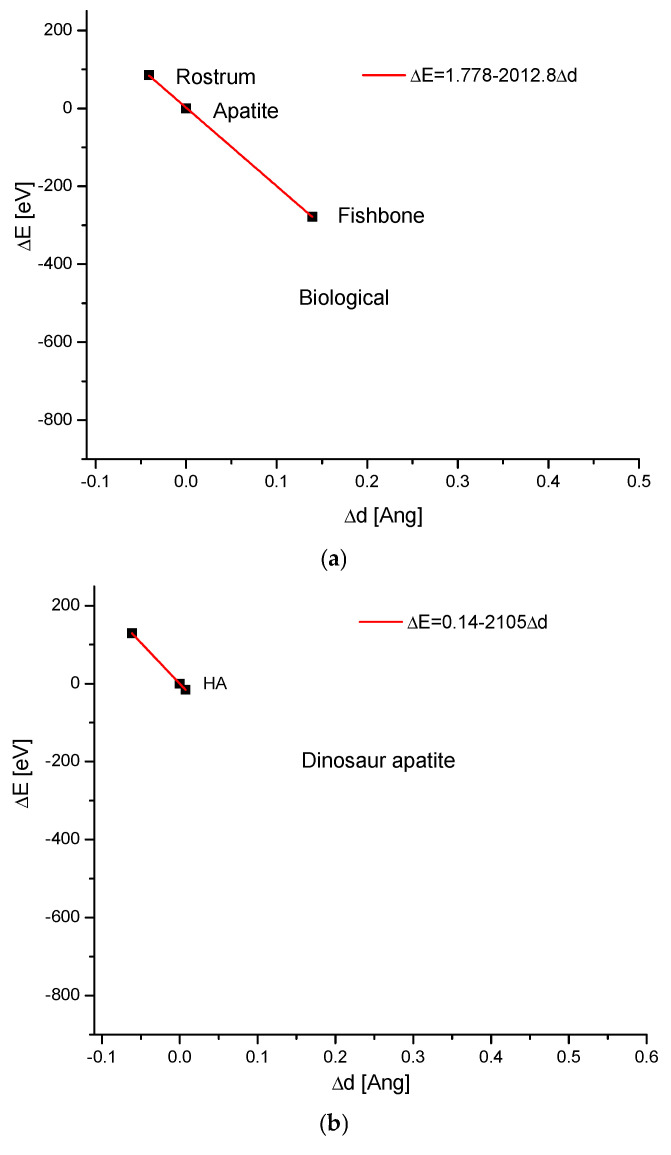

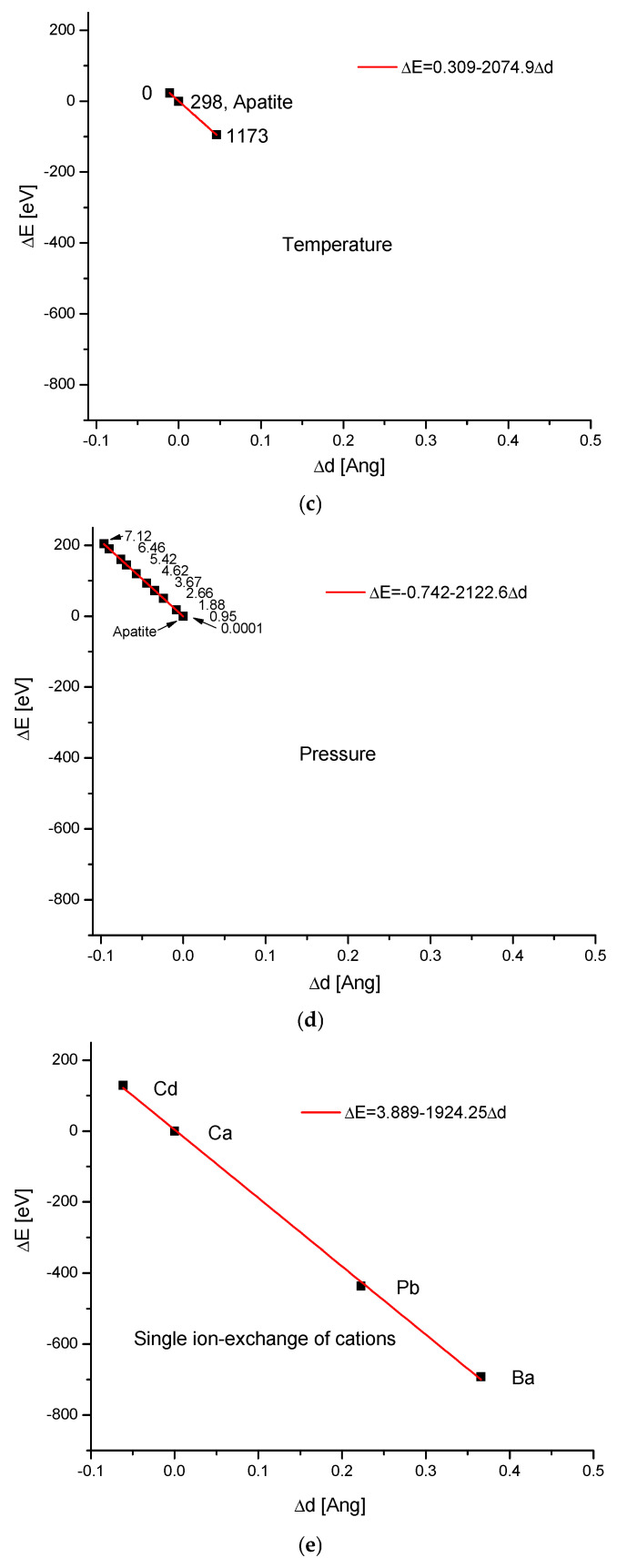

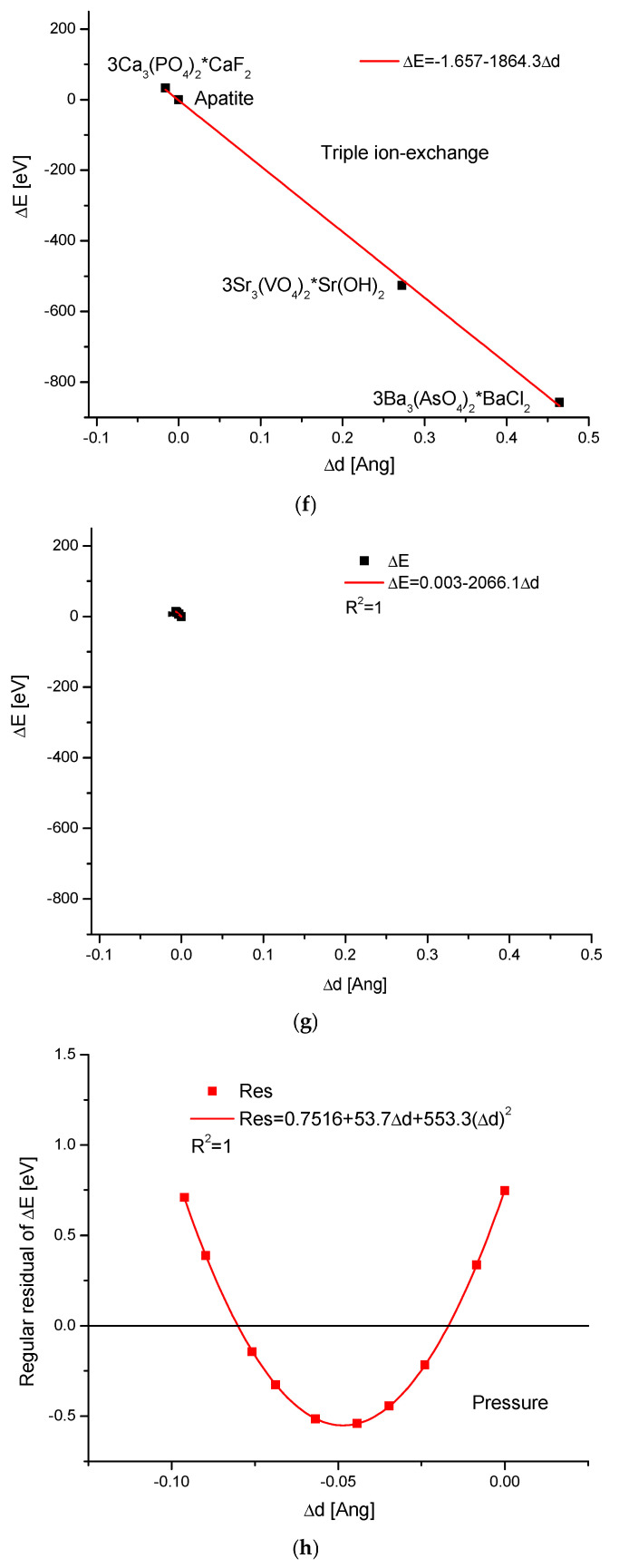
The ranges of apatite changes under the influences of (**a**) biological factors; (**b**) biological/geological factors in dinosaur fossils—the transformation into francolite; (**c**) temperature (Knyazev et al. [44]); (**d**) high pressure (Matsukage et al. [45]; somewhat similar data in Forien et al. [46]); (**e**) single-ion exchange; (**f**) total-ion exchange: all of the cations, the anions, and the channel species. The ranges of variables describing the axes of the diagrams are the same across figures (**a**–**g**) for clear comparison. The apatite point always indicates the hydroxyapatite standard, with Δd = 0 and ΔE = 0, from the assumption; (**g**) scattering of standard apatite measurements, according to the data from papers [47,48,49,50,51]; (**h**) the residuals of ΔE values from the approximation of the ΔE vs. Δd curve for the hydroxyapatite under high pressure (compare Figure (**d**)). See extraordinary regularity in the residual values.

The next problem to be solved is whether the range of apatite changes, as described in the framework of our formalism, ΔE–Δd diagrams, is unique only when it concerns biological actions. The range of biological variability was extracted from our data (Figure 1) and presented concisely in Figure 2a. It is somewhat surprising that the curve is asymmetric in relation to the hydroxyapatite standard. The organisms use rather less condensed forms of apatites. Figure 2b presents the data for the dinosaur fossil apatites, which are originally biological but are influenced by geological factors, including high pressure and transformation into francolite, a specific mineral containing both fluorine and carbonates, the monoclinic cousin of apatite [43]. The monoclinic form of francolite does not exclude it from the isomorphic series of apatites (observe the slopes of the curves). Nevertheless, the dinosaur bones exhibit a denser structure compared to standard hydroxyapatite.

For the comparison, the variability resulting from the heating is shown in Figure 2c. The influence of temperature is clear, but much narrower than that of biological actions. Here, the variability is one-sided: starting from the temperature 0 K, it always leads to swelling with increasing temperature. Obviously, the comeback is possible with the temperature drop, but only if the compound has not changed phase during the previous warming. Another case, evident in studies on the energy and dimensional changes in apatite crystals, relies on the pressure effect. Here (Figure 2d), the increase in pressure exerts a clear squeezing effect on the crystals.

To fully consider the allowed changes in the ΔE–Δd diagrams, we added new figures for the chemical changes, i.e., ion exchanges. The first one illustrates the case when only one type of cation is exchanged, leading to the synthesis of substituted compounds in the same crystallographic system (Figure 2e). We know from our earlier contribution that, among the three allowed kinds of ion exchange—of cations, anions, and simple chemical moieties—at the channel location, the first exerts the greatest effect. Indeed, the ranges of introduced changes in ΔE and Δd values are larger than in the previously shown cases. The next case confirms our suppositions about the prevalence of cationic exchanges—in Figure 2f, the complete exchanges involving the three allowed chemical entities are shown. The total change, although very significant, is not markedly different from that in a single cationic exchange. Undoubtedly, the ion exchange of all ions leads towards the synthesis of a totally new compound, but we—from our assumption—selected the substances organized in the same crystallographic system. Since measurements in different laboratories can be performed relative to different, locally determined hydroxyapatite standards, hydroxyapatite scattering is shown in Figure 2g. The data were taken from the classic papers mentioned in the caption. One can see that the deviations from Hughes’s results are small. This can be accounted for by a small shift along the ΔE–Δd curve. In addition, this correction is really important only for popular bioapatites. It is worth noting that the coefficient in the equation located in the figure is very close to that estimated in Equation (2), characteristic of the spontaneous expansion of the crystal cell.

Another statistical estimate of the robustness of the results relies on comparing the residuals obtained during the creation of the ΔE–Δd function, e.g., for the case when hydroxyapatite is pressurized (Figure 2h). It is an astonishing fact that the residuals, as such, also form an extremely regular structure. This seems to confirm our conviction that apatite variability is strictly quantitative.

In summary, we can state that there are the following ways to change crystal size under the influence of the factors presented in Table 1.

### 2.2. Passing to the Absolute Values

Now, we can pass to the absolute values of d, departing from the increments. One can ask, what happens to the volumes of the crystal cells of compounds if we pass from dimensions d(111) to the volumes? To answer this question, we will cite only three cases. The first one shows the relationships between V-d for the biominerals in Figure 4 (the reference to Figure 1a); the second one shows the relationship for hydroxyapatite under pressure (refer to Figure 2c). The third one presents the relationships connected with the ion exchange in cationic positions. The similar relationships concern all the previously considered cases.

The relationship is surprising and very simple and regular, which is not obvious if we consider the variability of parameters a and c for apatites, but more obvious if one takes into account that Bragg’s law concerns parameter d and not the ones previously mentioned:

V = K_3_ + K_4_d + K_5_d^2^ (4a)

In fact, the correction of the second order is significant only in some extreme cases, e.g., in the case of very radical full ion exchanges. In most applications, the relationship (5a) will be limited to the first two terms of the equation.

Since relationship (5a) acts also on the opposite side, we can transform Bragg’s law in a more illustrative way:

6.2 ∗ K_4_/E = (V – K_3_) ∗ sin θ (3b)

The theoretical value of V/d can be derived from the geometrical assumptions for the hexagonal system, where the volume of the cell is

V = √3 ∗ a^2^c/2 (4b)

and, from Equation (5),

(5) $$\frac{1}{d^{2}}=\frac{4}{3}\left(\frac{h^{2}+hk+k^{2}}{a^{2}}\right)+\frac{l^{2}}{c^{2}}$$

For the (111) orientation,

d = ac/√(a^2^ + 4c^2^) (4c)

Thus,

V/d = a/2√(3a^2^ + 12c^2^) (4d)

**Figure 3:**
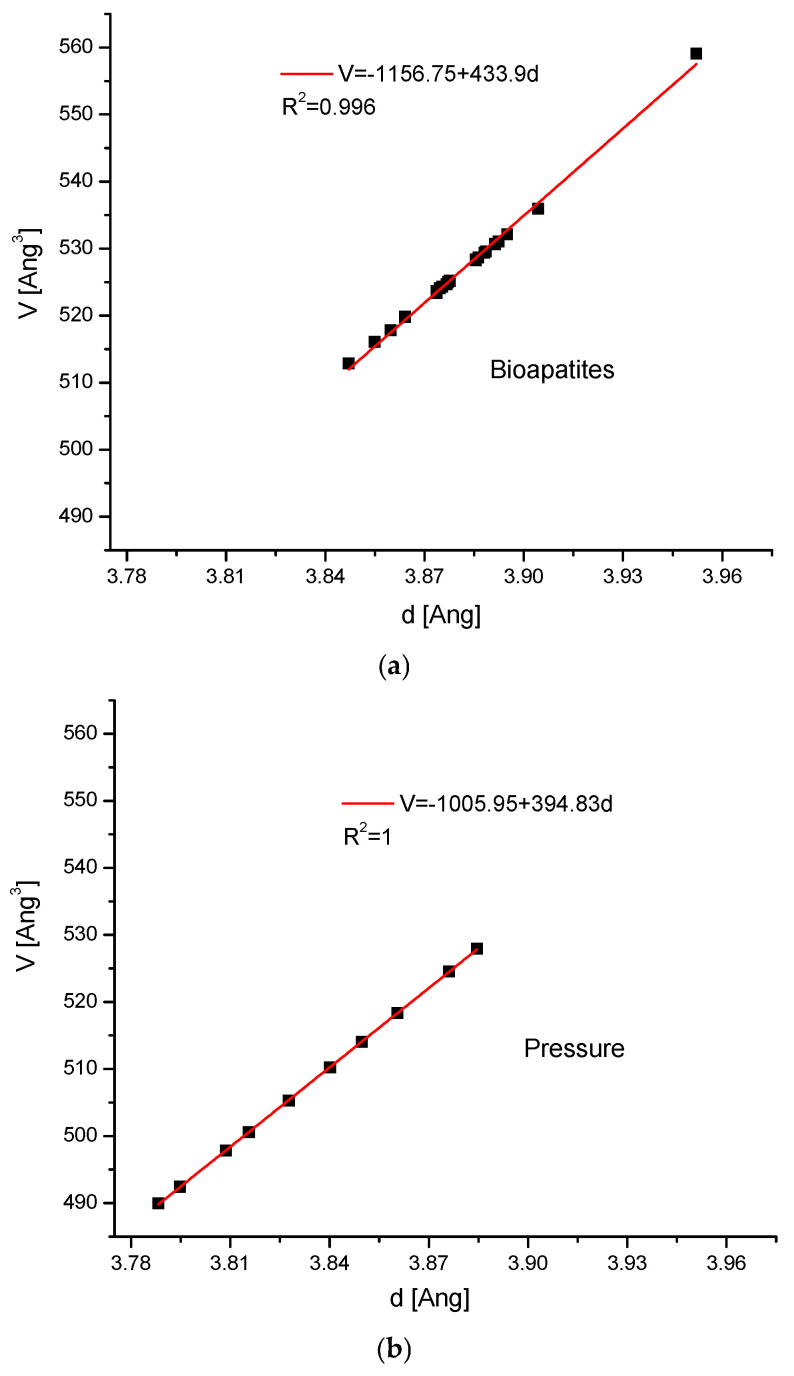

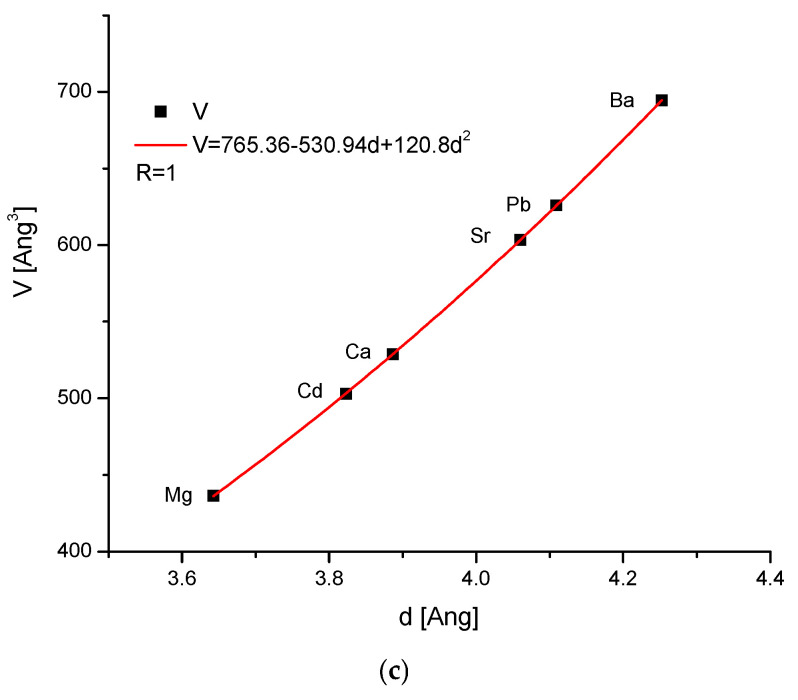
V/d relationships for: (**a**) bioapatites from Figure 1a; (**b**) pressurized hydroxyapatites from Figure 2c; (**c**) the series of hydroxyapatites with variable cations; compare with Figure 2e.

**Figure 4:**
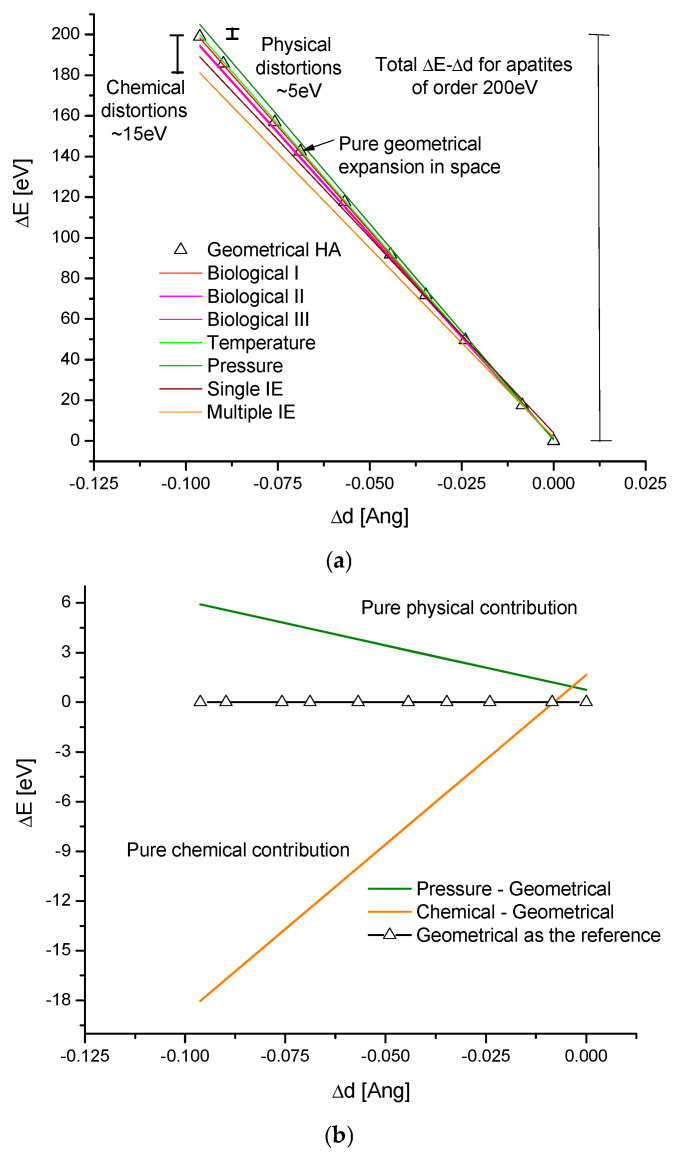
(**a**) The summary diagrams ΔE–Δd for different apatites and bioapatites as related to the methods of expansion of crystals. The lines in the tones of red show biological variability, the tones of green—physical effects, and the tones of brown—chemical ion exchanges. The line with the triangle means the basic geometrical expansion. The vertical lines denote the energetic distortions between different ways of expansion; (**b**) extracted pure physical and chemical contributions obtained by the subtraction of geometrical contributions.

After the introduction of the above-mentioned values of a and c for our standard hydroxyapatite, the result is 135.9, which is in very good accordance with the data from Figure 4: the values are 135.97 from Figure 3a, 135.89 from Figure 3b, and 135.71 from Figure 6c, respectively. This simplifies the formulation of Bragg’s law for pure hydroxyapatite:

6.2/E = 1/135.9 ∗ V ∗ sin θ(3c)

and, in fact,

842.58/E = V ∗ sin θ

where E is in keV, while V is in Å^3^.

Thus, Bragg’s law in such an approach as Equation (3b) (and in the simplified form (3c)) is an interplay between the apparent probing energy E and the available internal crystallographic cell volume V, controlled by the value of the sin of the angle of positive diffraction. This is a peculiar kind of geometry inside the crystal cell, where the volume is proportional to the Bragg’s dimension (here (111)). The energy is, from the assumption, the apparent probing energy, since its action is coupled to the increase/decrease in the crystal cell volume. Here, it is centred around the exciting energy of 8.041 keV for the Cu tube, but excitation using monochromatic lines from other types of X-ray tubes or synchrotron lines would form local environments around other lines. Thus, we can probe the crystals in many ways. This concerns some peculiar small spaces covering some hundreds of Å^3^ under pressures expressed in gigapascals (Figure 2d) and in the temperature range 0–1100 K (Figure 2c).

When we have the possibility of immediately determining the volume from X-ray diffraction measurements, we can use the instrument for studies in the field of volume-based thermodynamics.

## 3. Discussion

We managed to collect a large amount of X-ray diffraction data on the apatite molecules that constitute different organs of many animals and humans, as well as some defective excretions. The system was greatly expanded by the addition of data on various mineral apatites. We applied our earlier method of calculation for the apparent change in exciting energy in relation to the change in Bragg’s dimension d (mainly d(111)). Using the very fine linear results from Equation (1c), we proved that this equation can be supplemented by the equally simple Equation (4a), which gives the volume of the crystal cell inside. Then, Bragg’s equation can be transformed from a classical wave form into the E vs. V form in Equation (3b). In such a form, it does not immediately express the constructive diffraction phenomena, but the simple fact that each de/compression resulting in volume changes shows the apparent effect on the probing energy.

One can distinguish the geometrical, thermal, pressurized, biological, and ion-exchange-caused (chemical) volume expansions of isomorphic crystals. If we compare the coefficients of the slopes in the equations in Figure 2, some conclusions can be easily drawn.

- According to Equation (2), one can describe the geometrical expansion of the hexagonal system using the coefficients from the above equations, as if only the space expansion occurred.
- The thermal expansion (Figure 2c) is most similar to the geometrical expansion, and we can imagine that it results from the nonuniform distribution of the thermal effects within the crystal cell, which has a different symmetry from the spherical one, and the thermal heterogeneity is most tailored to this situation.
- The situation during the pressure action (Figure 2d) is more distant than in the geometrical case. This results from the fact that the uniform pressure exerts a nonuniform influence on ions located at different points in the hexagonal space, thus having a variable primary energetic status. At the margin, according to our intuition, the effects of temperature and pressure would be closer to each other if we worked with other crystals in the cubic crystallographic system. Both temperature and pressure actions are of physical character, and their additional contribution is approximately ~5 eV. They are reversible unless the conditions for the phase change are met.
- Much more pronounced are the chemical effects. We generally consider them as ion exchanges, with the single ones leading to the substituted compounds and with the multiple ones forming quite new compounds. We must only remember that we solely consider the substances belonging to one selected crystallographic class. Here, the numerous small distortions (asymmetries) arrive. The range depends on the sizes of the introduced ions relative to those of the original ions. In general, the distortions and overcoming the internal strains decrease the energies of the order to −15 eV. The chemical influence on crystal expansion is the greatest among all the considered factors. The chemical effects are sensitive to phase changes, as seen in the transition from hexagonal to monoclinic structure in chlorapatite.
- The biological apatites, whose variability results from limited ion exchanges and vacancies in response to specific ion supplies, lie relatively close to the geometrical trajectory of volume expansion; what is interesting is that it is possible to observe an approximate inversion of the synthesis processes in erosion/decay-type reactions [41].

All that was said above is summarized in Figure 4. The contributions were grouped by colour. From the sizes of physical (here pressure-invoked) and chemical (here multiple-ion exchanges) changes, the geometrical contributions (the reference values determined using Equation (2)) were subtracted. The physical and chemical influences were presented in a “naked” shape (Figure 4b). This is shown in the diagrams of the ΔE–Δd type.

### 3.1. Intracrystalline Approach

Up to now, we have mainly concentrated on the questions of the growth increments of the essential quantities: E—the apparent probing energy, d—Bragg’s distance, and V—the volume of the crystal cell. Now, similarly to Figure 4 and Equations (3) and (4), some attention must be paid to the absolute values. The probing energy value can be easily established, since it is simply 8.041 + ΔE [keV] from the assumption. The value of 8.041 keV corresponds to the photon energy of the essential K lines in the spectrum of the Cu tube. It establishes the first level, from which we calculate the next levels by adding the corrections ΔE. One must remember that the E value is the apparent probing energy from the selected excitation source. Perhaps the values of ΔE have greater practical meaning, since they can probably reflect the real difference in energy levels between different compounds or between different states of the selected compound, and are relatively easy to experimentally verify, e.g., using calorimetric methods. However, one cannot exclude that their meaning is only theoretical and ordering in relation to isomorphic compounds.

The solution is quite obvious if one considers Equation (1c) as the differential version of another more general equation expressed in absolute values for hydroxyapatite and takes into account the relationship in Equation (4d), which leads immediately to the formulation

**Figure 5:**
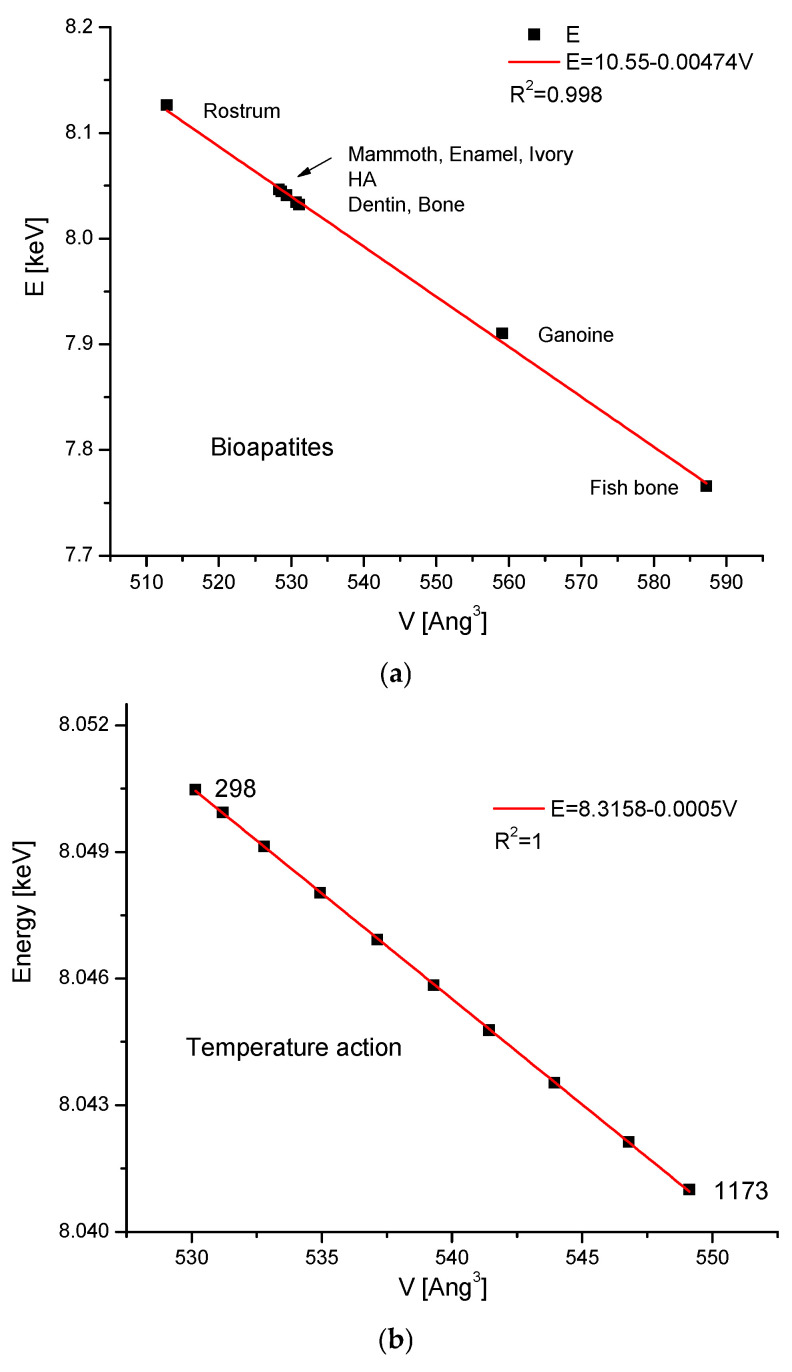

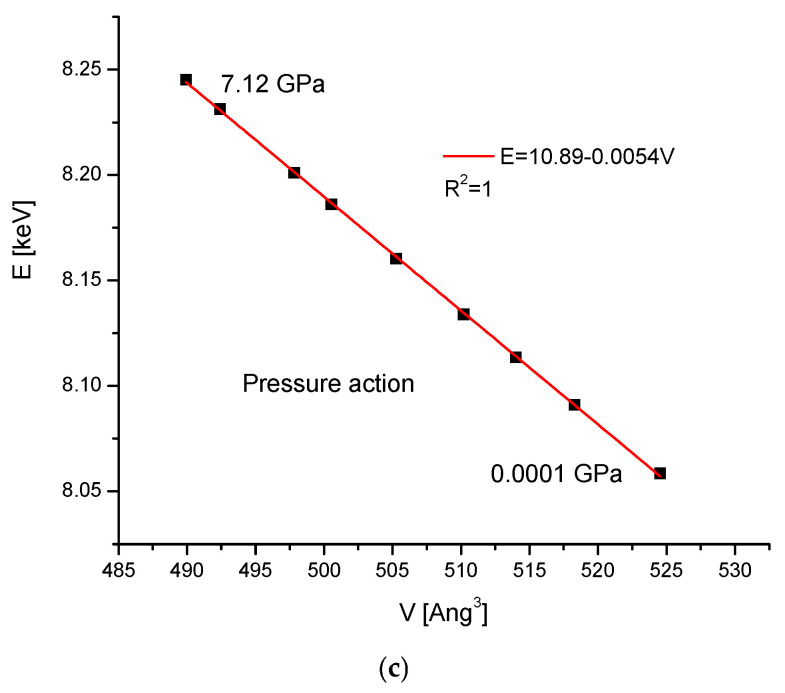
The elegant functional dependence between the total probing energy of crystal compression and the volume of the crystallographic cell: (**a**) for bioapatites; (**b**) for temperature-invoked changes (related to Figure 2b); (**c**) for pressure-invoked changes (related to Figure 2c). Please note that Figure 6 is the analogue of differential Figure 2 in the absolute values of E and V.

E = K_5_ − K_6_ V (6)

and gives a particularly simple relationship between the absolute values of E and V, not just between the increments. This is clearly shown in Figure 5. Equation (6) is not the basic equation. There is Equation (1), which is rigorously derived, while Equation (6) is the empirically derived integral equivalent of Equation (1). Equation (6) is indirectly drawn from Equation (1c), and its accuracy depends on how real dimensions a and c interplay, giving the value d(111).

However, joining Equations (3c’) and (6), we can obtain the especially simple version of Braggs’ law:

K_7_/sin θ = V(K_5_ − K_6_V) (3e)

where one of the variables, the apparent probing energy E, is eliminated. It is the second-order equation of sin θ against V. The measurement of the diffraction angle yields the crystal cell volume as one of the two roots of this equation.

The unique method of calculating crystal cell volumes directly from X-ray diffraction measurements promises to make it easier to obtain the values necessary for thermodynamic measurements [53].

### 3.2. Interpretation

The irradiation of the sample allows the probe to measure the new equilibrium in the crystal cell. The initial conditions are as follows: the exciting energy is equal to 8.041, and the volume of the standard hydroxyapatite cell is 527.9Å^3^. Each deviation from the assumed volume results in a change in the apparent probing energy, since this energy is tailored to the real volume of the crystal cell. The probing energy and the cell volume are coupled by Equations (3b,c) (transformed Bragg’s equation) and (6). For more explanations—see Appendix A.

### 3.3. Deviations

As shown in Figure 5a, there is clear scatter in the curves which formally present the different isomorphic series, with hydroxyapatite as the member. In cases where the parameter k in Equation (1c) is close to −2068.9, we have the linear versions of Equations (1c) and (6). However, the extensive ion exchanges lead to greater deviations in the value of k. We can consider the case in which Ca in hydroxyapatite is fully substituted by divalent cations, ranging from Mg [54] to Ba.

We know that other researchers did not confirm Patel’s claims regarding the possibility of the full synthesis of Mg-apatite. Nevertheless, when we include Patel’s data in our calculations, we obtain perfect results, as if such a compound indeed exists. We decided to include the relevant data in our diagrams, with the full awareness that it may be only a result of a mathematical trick. Similarly, Drouet [55] used data on the magnesium compound to obtain quite credible data on the thermodynamic functions for MgHA.

**Figure 6:**
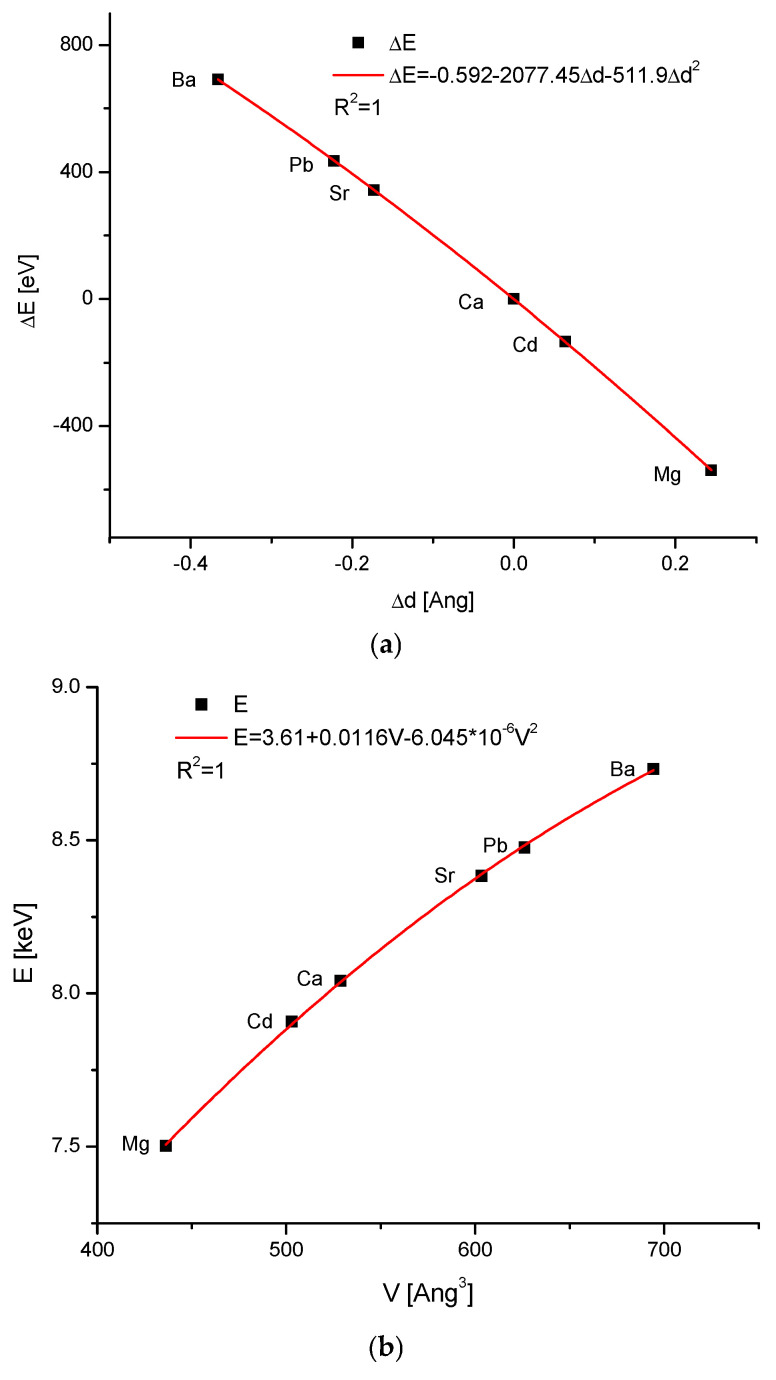

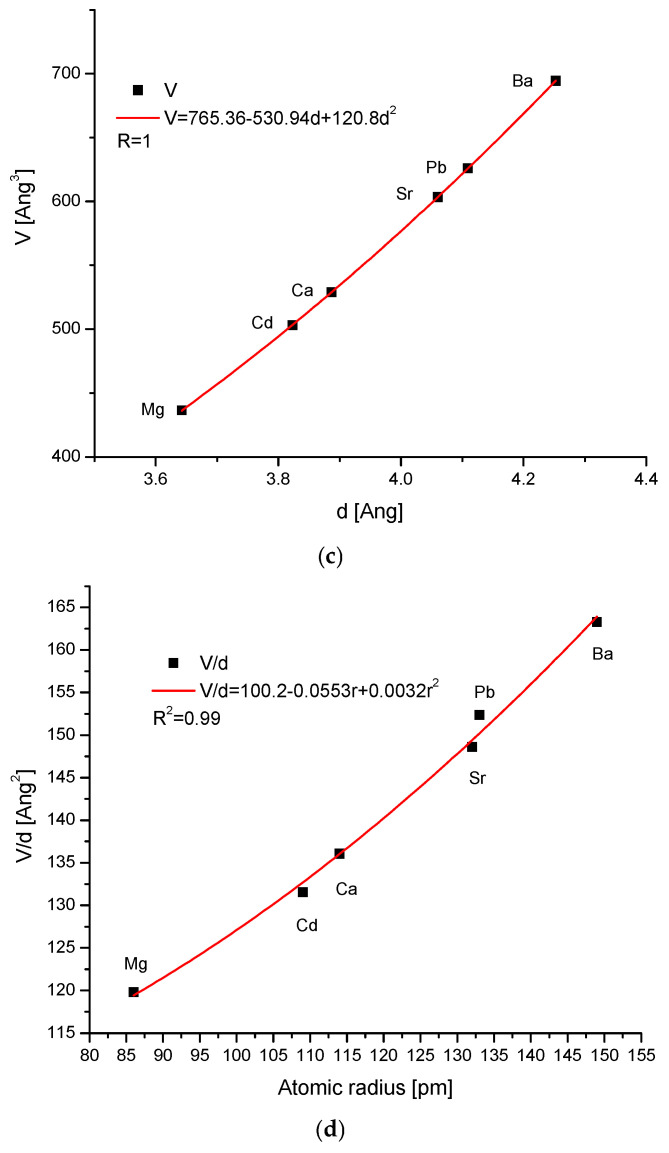
(**a**) ΔE–Δd diagram for the isomorphic series of hydroxyapatites substituted with the consecutive cations; (**b**) the same system in the absolute values E-V; (**c**) V-d relationship; (**d**) V/d value as strictly dependent on the ionic radius.

The cases with a partial substitution (e.g., Fe, Co, Mn, and others) are not taken into account here. One can observe that even the basic curve of ΔE–Δd is slightly deviated from linearity (Figure 6a). Nevertheless, the curve is perfectly approximated with a polynomial of the second order. Moreover, the corrections in terms of first- and second-order are small, and one can observe that the essential coefficient −2077.45 is not far from the theoretical value of −2068.9. It does not prevent participating in the mentioned elements in one isomorphic series. The nonlinearity is reflected in the version expressed in the absolute values (Figure 6b), i.e., in the function E–V. The relationship expressing the V–d curve is also a function of the second order (Figure 6c). Now, we can pass to another relationship. If we consider the ionic radii [56] of the ion substituting Ca in the structure of the hydroxyapatite, then it is clear that the cations influence the volume of the crystal cell, to which they enter. In such a situation, we cannot treat the apatite cells as the invariable units, since they simply adjust their dimensions to the new elements (Figure 6c,d). This was anticipated theoretically by Wu et al. [57].

## 4. Materials and Methods

### 4.1. Materials

For the data search, we used platforms and databases such as Web of Science, Scopus, Scilit, and Google Scholar. We used available data from the world literature on widely understood apatite-type compounds with hexagonal structure, and X-ray, electron, or neutron diffraction [58] were the only instrumental methods considered for their description. The fine presentation of what can be determined using X-ray diffraction was given by Moulick et al. [59]. The hexagonal structure is considered only for the uniformity of the results and is not unique. Where it is necessary, we consider a monoclinic structure as well, after a small recalculation. Identical reasoning can be applied to other, but exclusively uniform, crystallographic classes of substances. No experiment considered in this paper was performed in the laboratories of the authors, which does not mean that they do not conduct experiments; they also conduct experiments in the field of X-ray diffraction [60,61], sometimes using very advanced X-ray 2D imaging techniques [62]. Simply, nobody in the world had the potential to make all the demanded measurements. The data are supported by the sources, which are sometimes well documented when they concern human enamel, dentin and bone, while they are much scarcer when they concern ivories, antlers, or rostra. It was a question of proper selection among the numerous data in the former case and of utilizing the available data in the latter case. Pure inorganic apatites were considered in even more detail. The special inspiration could be found in the papers [55,57,63,64,65,66,67,68]. The results were presented in the form of ΔE–Δd diagrams described by Equation (1) or other similar ones where it was relevant. The values of the ΔE and Δd parameters were always determined relative to hydroxyapatite values; thus, most results were relative. As a rule, the values of Hughes et al. [48] were applied in our contribution for this compound, and are presented in Table 2.

The dimensions d are given in angstroms Å, temperatures T as the absolute Kelvin K, and pressures P in gigapascals GPa. The results of ΔE calculations are then reported in kiloelectronvolts, but they are often recalculated to electron volts eV in our contribution.

The authors of the databases are appropriately cited in the text, and we are deeply indebted to them. Due to the huge amount of data, we could not clearly describe all the bioapatites in Figure 1a, and we decipher the particular numbers in the caption.

### 4.2. Methods

Supplementing previously cited equations, we should write Bragg’s equation in the energetic representation [69]:

12.4/E = 2dsin θ(3a)

and add the interrelations between Miller indices in the hexagonal system:

1/d^2^ = 4(h^2^ + hk + k^2^)/ 3a^2^ + l^2^/c^2^ (5)

After introducing the relevant data for our hydroxyapatite standard [13], we found the theoretical values of K_1_ −2068.6eV/Å from Equation (2a) and K_2_ −2069.2eV/Å from Equation (2b). The good approximation of the constant k in Equation (1c) is the value −2068.9, and it expresses the ideal model of an isomorphic series if its members expand only due to geometrical reasons.

Since we have many coefficients in our equations, we denoted them all with K letters, each with a corresponding consecutive subscript. All the equations in our contribution, except Equations (3a) and (5), are derived by the authors. The equations of special importance are presented in bold.

Please note that the coefficients of determination R^2^ in our figures, in the majority of cases, have values of 1 or very close to 1, indicating that our equations are of functional character and not merely approximations of the experimental results. Otherwise, in many cases, the equations are rigorously derived from earlier assumptions.

The authors paid special attention to robustness and the self-tailoring of the results. At first, selecting the available results was necessary. Sometimes it was simple due to the scarcity of the materials (as for the whale rostrum); in other cases (e.g., human bones), we had to make the selection, and then the laboratory basis, the description of the experiment (Knyazev et al. [44], Matsukage et al. [45]), or the multiple uses in the scientific literature (Hughes et al. [48] for the standards) were the decisive reasons. The authors who reported a whole series of coincident results were preferred (Piga et al. [37,38]). All the deviations in the Δd value were repeated for the ΔE values due to the construction of Equation (1a–c). So, for the uniform sets of results, the local deviations were meaningless. Values very close to 1 for the coefficient of determination R^2^ result from the rigorousness of the derivation of Equation (1), and they are confirmed by the astonishing results shown in Figure 2h.

The procedure of the calculations for the construction of ΔE–Δd diagrams is very simple:

- Using the values of parameters a and c, calculate d according to Equation (4);
- Subtract the values d from d_0_ (standard), getting Δd;
- Using the latter value, calculate ΔE according to Equation (1a) or (1b);
- Furthermore, it is a trivial arrangement of the variables in the diagrams.

The paper, although based exclusively on the studies of other researchers, is absolutely original in the concepts of the authors and, to our knowledge, has no analogue in the world’s literature.

### 4.3. Software

Origin 9.1 was used for result evaluations and graph preparation.

## 5. Conclusions

We collected available data on apatite crystals in very different biostructures, including the extreme cases—whale rostrum and ganoine. The expansion of the crystallographic cells in bioapatites was compared with processes of expansion induced by thermal and pressure actions and by ion exchange, whether of single ions (substitutions) or of all ions (synthesis of a new compound). The condition was that all ΔE–Δd diagrams should concern the compounds existing in the same crystallographic system. Essentially, all the studied diagrams were very similar with respect to the parameters describing the relevant straight lines. The wide possibility of steering with the crystal cell volume was proven. We extracted subtle differences in the slopes of the lines and attributed them to differences in the physical or chemical factors reflected in the ΔE–Δd diagrams. The discoveries could be transmitted into absolute values, with the absolute probing energy E = 8.041 + ΔE if the excitation was with the Cu X-ray tube and V = K_3_ + K_4_d, or alternatively V = √3 * a^2^c/2 for the hexagonal system in configuration (111). Once the relationship between the Braggs’ dimension d and volume V was established, we proposed a new transformed version of the generalized Bragg’s law, 6.2 * K_4_ /E = (V − K_3_) * sin θ, and even a simplified version, k_7_/E = V * sin θ, expressed only in mechanical variables.

## Figures and Tables

**Table 1:** Influence of different factors on the change in the sizes of crystals.

Factor	Hypothesized Ways of Changing the Sizes of Crystals
Simple geometrical extension	Described by the values of coefficients K from Equation (2), here −2068.9 for the hydroxyapatite series
Temperature	Typical physical factor, up to the phase transition or decay
Pressure	Another physical factor, up to the deformation, the phase transition or the crashing
Single-ion exchange	Leads to the synthesis of substituted compounds, chemical action
Multiple-ion exchange	Leads to the synthesis of quite new compounds, chemical action
Biological factors [52]	Can involve small-ion exchanges, controlled substrate supplies, controlled space limitations inside organic matrices
Biological joined with geological factors, as in fossils	Leads to some chemical and crystallographic changes, here to the formation of francolites

**Table 2:** Crystal parameters.

Crystal Parameter	Value
a	9.4166 [Å]
c	6.8745 [Å]
V	527.9 [Å^3^]

## Data Availability

All data were presented and/or referenced.

## Appendix A

### Possible Interpretation of the Apparent Exciting Energy

We try to explain the meaning of E and ΔE in our reasoning. At first, the real exciting energy E_0_ (in this case 8.041 keV from Cu tube) represents in fact the invariable value. Δd means the change in the interplanar distance d and is real, in the same way as the value of sin θ. ΔE is the imaginary value, resulting from Equation (1). The results of measurements in our approach are always relative; they are, as a rule, compared to pure hydroxyapatite. Our reasoning is supported by the idea that we aim to transform hydroxyapatite in any way—physical, chemical, or biological.

The values of ΔE are quite significant. If we assume that we often meet in our data Δd = 0.01Å, then we have ΔE = −20.689 eV; similarly, for Δd = 0.1Å, we have ΔE = −206.89 eV. Those energies correspond to radiation wavelengths in the range of ~1–100 nm, i.e., corresponding to very soft X-rays–extreme UV. We can treat ΔE conventionally as a disturbance in lattice oscillations in such an energy range.

When we consider absolute values, we must assume that E is E_0_ + ΔE. This means that E is the apparent Stokes (when ΔE is a negative quantity) or the anti-Stokes quantity. In the first case, we are dealing with decompression, while, in the second case, we are dealing with cell compression. Such an approach to the E value resembles the quasi-Raman phenomenon, of course, the imaginary one in this case.

Such an interpretation seems to us to have a considerable probability.

## References

[1] Nasoori A. (2020). Formation, Structure, and Function of Extra-skeletal Bones in Mammals. Biol. Rev..

[2] Rogers K.D., Zioupos P. (1999). The Bone Tissue of the Rostrum of a Mesoplodon Densirostris Whale: A Mammalian Biomineral Demonstrating Extreme Texture. J. Mater. Sci. Lett..

[3] Tang L., Zhang L., Yue M., Tian D., Su M., Li Z. (2019). New Insights into the Ultrastructure of Bioapatite After Partial Dissolution: Based on Whale Rostrum, the Densest Bone. Microsc. Microanal..

[4] Elliott J.C. (2002). Calcium Phosphate Biominerals. Rev. Mineral. Geochem..

[5] Forien J.-B., Zizak I., Fleck C., Petersen A., Fratzl P., Zolotoyabko E., Zaslansky P. (2016). Water-Mediated Collagen and Mineral Nanoparticle Interactions Guide Functional Deformation of Human Tooth Dentin. Chem. Mater..

[6] Habraken W., Habibovic P., Epple M., Bohner M. (2016). Calcium Phosphates in Biomedical Applications: Materials for the Future?. Mater. Today.

[7] Schultze H.P. (2016). Scales, Enamel, Cosmine, Ganoine, and Early Osteichthyans. Comptes Rendus Palevol.

[8] Kawasaki K., Keating J.N., Nakatomi M., Welten M., Mikami M., Sasagawa I., Puttick M.N., Donoghue P.C.J., Ishiyama M. (2021). Coevolution of Enamel, Ganoin, Enameloid, and Their Matrix SCPP Genes in Osteichthyans. iScience.

[9] Šupová M. (2014). Isolation and Preparation of Nanoscale Bioapatites from Natural Sources: A Review. J. Nanosci. Nanotechnol..

[10] Dal Sasso G., Asscher Y., Angelini I., Nodari L., Artioli G. (2018). A Universal Curve of Apatite Crystallinity for the Assessment of Bone Integrity and Preservation. Sci. Rep..

[11] Currey J.D. (2004). Tensile Yield in Compact Bone Is Determined by Strain, Post-Yield Behaviour by Mineral Content. J. Biomech..

[12] Pasteris J.D., Wopenka B., Valsami-Jones E. (2008). Bone and Tooth Mineralization: Why Apatite?. Elements.

[13] Dorozhkin S.V. (2016). Calcium Orthophosphates (CaPO_4_): Occurrence and Properties. Prog. Biomater..

[14] Pasteris J.D., Wopenka B., Freeman J.J., Rogers K., Valsami-Jones E., van der Houwen J.A.M., Silva M.J. (2004). Lack of OH in Nanocrystalline Apatite as a Function of Degree of Atomic Order: Implications for Bone and Biomaterials. Biomaterials.

[15] Li Z., Pasteris J.D., Novack D. (2013). Hypermineralized Whale Rostrum as the Exemplar for Bone Mineral. Connect. Tissue Res..

[16] Akkus A., Karasik D., Roperto R. (2017). Correlation between Micro-Hardness and Mineral Content in Healthy Human Enamel. J. Clin. Exp. Dent..

[17] Kuczumow A., Gorzelak M., Kosiński J., Lasota A., Szabelska A., Blicharski T., Gągała J., Wawrzyniak J., Jarzębski M., Jabłoński M. (2023). Quantitative Description of Isomorphism in the Series of Simple Compounds. Int. J. Mol. Sci..

[18] Kuranov G., Nikolaev A., Frank-Kamenetskaya O., Gulyaev N., Volina O. (2019). Physicochemical Characterization of Human Cardiovascular Deposits. JBIC J. Biol. Inorg. Chem..

[19] Miake Y., Yamazaki T., Yanagisawa T., Sakae T., Suwa T., Okazaki M. (2009). Morphological and Crystallographic Properties of Tooth Enameloid of Medaka (*Oryzias latipes*). J. Hard Tissue Biol..

[20] Dorozhkin S.V. (2006). Calcium Phosphates and Human Beings. J. Chem. Educ..

[21] Ayliffe L.K., Chivas A.R., Leakey M.G. (1994). The Retention of Primary Oxygen Isotope Compositions of Fossil Elephant Skeletal Phosphate. Geochim. Cosmochim. Acta.

[22] Sun X., He M., Wu J. (2022). Crystallographic Characteristics of Inorganic Mineral in Mammoth Ivory and Ivory. Minerals.

[23] LeGeros R.Z., Contiguglia S.R., Alfrey A.C. (1973). Pathological Calcifications Associated with Uremia. Calcif. Tissue Res..

[24] Mandel I., Mandel N. (2007). Structure and Compositional Analysis of Kidney Stones. Urinary Stone Disease.

[25] Stork L., Müller P., Dronskowski R., Ortlepp J.R. (2005). Chemical Analyses and X-Ray Diffraction Investigations of Human Hydroxyapatite Minerals from Aortic Valve Stenoses. Z. Krist. Cryst. Mater..

[26] Rouzière S., Bazin D., Daudon M. (2016). In-Lab X-Ray Fluorescence and Diffraction Techniques for Pathological Calcifications. Comptes Rendus Chim..

[27] Chen I.H., Yang W., Meyers M.A. (2014). Alligator Osteoderms: Mechanical Behavior and Hierarchical Structure. Mater. Sci. Eng. C.

[28] Fink J., Tremblay M.M., Tobin T.S., Stockli L.D., Stockli D.F., Ickert R.B. (2024). Diagenesis of Fossil Gar Fish Scales with Implications for Geochronology and Paleoenvironmental Applications. Geochim. Cosmochim. Acta.

[29] Chen P.Y., Stokes A.G., McKittrick J. (2009). Comparison of the Structure and Mechanical Properties of Bovine Femur Bone and Antler of the North American Elk (*Cervus elaphus canadensis*). Acta Biomater..

[30] González-Rodríguez L., López-Álvarez M., Astray S., Solla E.L., Serra J., González P. (2019). Hydroxyapatite Scaffolds Derived from Deer Antler: Structure Dependence on Processing Temperature. Mater. Charact..

[31] Enax J., Prymak O., Raabe D., Epple M. (2012). Structure, Composition, and Mechanical Properties of Shark Teeth. J. Struct. Biol..

[32] Kuhs W.F., Sänger A.T. (1992). Structural Disorder in Hydroxyapatite. Z. Krist. Cryst. Mater..

[33] Rodríguez-Lorenzo L.M., Hart J.N., Gross K.A. (2003). Structural and Chemical Analysis of Well-Crystallized Hydroxyfluorapatites. J. Phys. Chem. B.

[34] Boutinguiza M., Pou J., Comesaña R., Lusquiños F., De Carlos A., León B. (2012). Biological Hydroxyapatite Obtained from Fish Bones. Mater. Sci. Eng. C.

[35] Shi P., Liu M., Fan F., Yu C., Lu W., Du M. (2018). Characterization of Natural Hydroxyapatite Originated from Fish Bone and Its Biocompatibility with Osteoblasts. Mater. Sci. Eng. C.

[36] Kuczumow A., Blicharski T., Gorzelak M., Kosiński J., Lasota A., Gągała J., Nowak J., Jarzębski M., Jabłoński M. (2022). Measurements of Energetic States Resulting from Ion Exchanges in the Isomorphic Crystals of Apatites and Bioapatites. Molecules.

[37] Piga G., Santos-Cubedo A., Moya Solà S., Brunetti A., Malgosa A., Enzo S. (2009). An X-Ray Diffraction (XRD) and X-Ray Fluorescence (XRF) Investigation in Human and Animal Fossil Bones from Holocene to Middle Triassic. J. Archaeol. Sci..

[38] Piga G., Santos-Cubedo A., Brunetti A., Piccinini M., Malgosa A., Napolitano E., Enzo S. (2011). A Multi-Technique Approach by XRD, XRF, FT-IR to Characterize the Diagenesis of Dinosaur Bones from Spain. Palaeogeogr. Palaeoclim. Palaeoecol..

[39] Carlström D., Glas J.-E. (1959). The Size and Shape of the Apatite Crystallites in Bone as Determined from Line-Broadening Measurements on Oriented Specimens. Biochim. Biophys. Acta.

[40] Xie B., Nancollas G.H. (2010). How to Control the Size and Morphology of Apatite Nanocrystals in Bone. Proc. Natl. Acad. Sci. USA.

[41] Weiner S., Wagner H.D. (1998). THE MATERIAL BONE: Structure-Mechanical Function Relations. Annu. Rev. Mater. Sci..

[42] Lasota A., Gorzelak M., Turżańska K., Kłapeć W., Jarzębski M., Blicharski T., Pawlicz J., Wieruszewski M., Jabłoński M., Kuczumow A. (2024). The Ways of Forming and the Erosion/Decay/Aging of Bioapatites in the Context of the Reversibility of Apatites. Int. J. Mol. Sci..

[43] Zapata F., Roy R.N. (2004). Use of Phosphate Rocks for Sustainable Agriculture.

[44] Knyazev A.V., Chernorukov N.G., Bulanov E.N. (2012). Apatite-Structured Compounds: Synthesis and High-Temperature Investigation. Mater. Chem. Phys..

[45] Matsukage K.N., Ono S., Kawamoto T., Kikegawa T. (2004). The Compressibility of a Natural Apatite. Phys. Chem. Miner..

[46] Forien J.-B., Fleck C., Krywka C., Zolotoyabko E., Zaslansky P. (2015). In Situ Compressibility of Carbonated Hydroxyapatite in Tooth Dentine Measured under Hydrostatic Pressure by High Energy X-Ray Diffraction. J. Mech. Behav. Biomed. Mater..

[47] Kim J.Y., Fenton R.R., Hunter B.A., Kennedy B.J. (2000). Powder Diffraction Studies of Synthetic Calcium and Lead Apatites. Aust. J. Chem..

[48] Hughes J.M., Cameron M., Crowley K.D. (1989). Structural Variations in Natural F, OH, and Cl Apatites. Am. Mineral..

[49] Posner A.S., Perloff A., Diorio A.F. (1958). Refinement of the Hydroxyapatite Structure. Acta Crystallogr..

[50] Sudarsanan K., Young R.A. (1969). Significant Precision in Crystal Structural Details. Holly Springs Hydroxyapatite. Acta Crystallogr. B.

[51] Schwarz H. (1967). Verbindungen Mit Apatitstruktur. III. Apatite Des Typs M(X_VI_O_4_)^3^F_2_(M^II^ = Sr, Pb; X^VI^ = S, Cr; X^IV^ = Si, Ge). Z. Anorg. Allg. Chem..

[52] Foley B., Greiner M., McGlynn G., Schmahl W.W. (2020). Anatomical Variation of Human Bone Bioapatite Crystallography. Crystals.

[53] Puzio B., Manecki M. (2022). The Prediction Method for Standard Enthalpies of Apatites Using the Molar Volume, Lattice Energy, and Linear Correlations from Existing Experimental Data. Contrib. Mineral. Petrol..

[54] Patel P.N. (1980). Mangnesium Calcium Hydroxylapatite Solid Solutions: Preparation, IR and Lattice Constant Measurements. J. Inorg. Nucl. Chem..

[55] Drouet C. (2015). A Comprehensive Guide to Experimental and Predicted Thermodynamic Properties of Phosphate Apatite Minerals in View of Applicative Purposes. J. Chem. Thermodyn..

[56] Glasser L., Jenkins H.D.B. (2008). Internally Consistent Ion Volumes and Their Application in Volume-Based Thermodynamics. Inorg. Chem..

[57] Wu P., Zeng Y.Z., Wang C.M. (2004). Prediction of Apatite Lattice Constants from Their Constituent Elemental Radii and Artificial Intelligence Methods. Biomaterials.

[58] Tadano S., Giri B. (2011). X-Ray Diffraction as a Promising Tool to Characterize Bone Nanocomposites. Sci. Technol. Adv. Mater..

[59] Prosad Moulick S., Sahadat Hossain M., Zia Uddin Al Mamun M., Jahan F., Farid Ahmed M., Sathee R.A., Sujan Hossen M., Ashraful Alam M., Sha Alam M., Islam F. (2023). Characterization of Waste Fish Bones (*Heteropneustes fossilis* and *Otolithoides pama*) for Photocatalytic Degradation of Congo Red Dye. Results Eng..

[60] Kuczumow A., Chevallier P., Dillmann P., Wajnberg P., Michał R. (2000). Investigation of Petrified Wood by Synchrotron X-Ray Fluorescence and Diffraction Methods. Spectrochim. Acta Part B Spectrosc..

[61] Kuczumow A., Pikus S., Un-Ro C., Sadowski P., Wajnberg P., Jurek M. (2001). Structural Investigations of a Series of Petrified Woods of Different Origin. Spectrochim. Acta Part B Spectrosc..

[62] Kuczumow A., Nowak J., Kuzioła R., Jarzębski M. (2019). Analysis of the Composition and Minerals Diagrams Determination of Petrified Wood. Microchem. J..

[63] Pan Y., Fleet M.E. (2002). Compositions of the Apatite-Group Minerals: Substitution Mechanisms and Controlling Factors. Rev. Mineral. Geochem..

[64] White T. (2005). Apatite—An Adaptive Framework Structure. Rev. Mineral. Geochem..

[65] White T.J., ZhiLi D. (2003). Structural Derivation and Crystal Chemistry of Apatites. Acta Crystallogr. B.

[66] Drouet C. (2019). Applied Predictive Thermodynamics (ThermAP). Part 2. Apatites Containing Ni^2+^, Co^2+^, Mn^2+^, or Fe^2+^ Ions. J. Chem. Thermodyn..

[67] Flora N.J., Yoder C.H., Jenkins H.D.B. (2004). Lattice Energies of Apatites and the Estimation of Δ *H*_f_^°^(PO_4_^3−^, g). Inorg. Chem..

[68] Hughes J.M., Rakovan J.F. (2015). Structurally Robust, Chemically Diverse: Apatite and Apatite Supergroup Minerals. Elements.

[69] Lasota A., Gorzelak M., Bis E., Biliński P., Gieburowski K., Kłapeć W., Tymczyna-Borowicz B., Łobacz M., Pawlicz J., Jarzębski M. (2025). Implications of Isomorphism in the Family of Apatite Compounds. Int. J. Mol. Sci..

